# A U. S. Patent on Antitoxin

**Published:** 1898-09

**Authors:** 


A U. S. PATENT ON ANTITOXIN.--The medical profession of America have been startled and shocked at the announcement, recently published, that the U. S. Commission of Patents, after having refused the application *five times*, has at last issued to Behring, of Germany, a patent on the well-known and generally used process of making diphtheria antitoxin.

This action of the Patent Office is universally condemned; and is inexplicable. It is an outrage, and its injurious effects will be far reaching, and will cost the lives of many thousands of children throughout America. It is a prohibition put upon the only known remedy for and prophylactic of that dread scourge of children, diphtheria--a remedy which in its effects can only be compared to vaccine as a protection against smallpox, but having the inestimable advantage in that it is almost a specific in the *cure* of diphtheria. Any physician who now uses the remedy without Mr. Behring's permission—and without paying whatever price for it that his sweet conscience may dictate—is liable to prosecution in the courts of America. The children must die of diphtheria, as heretofore; Mr. Behring has willed it—unless the profession of American will pay the ransom. The great United States of America, through its great Commissioner, who has been induced by some means to change his mind, has put this injunction and the fate of our innocents entirely in the hands of this foreigner, who, by-the-by, is not the discoverer of antitoxin, nor is he the perfector of the process of manufacture—he having been preceded many years by Pasteur, Roux, Kitasato and others, and he simply appropriated their discovery and methods.

The patenting of a principle is not lawful—the process can only be so protected. The principal of serum therapy belongs to the medical profession; the method of preparation of the antitoxin in question is credited to Roux, I believe, and Mr. Behring or any other man would as justly be entitled, should he claim it, to a patent on Jenner's method of getting vaccine virus through the cow, or, indeed, upon the process of manufacturing chloroform!

It would seem to us that an official in the exercise of so powerful a prerogative as that of granting patents was either hasty and inconsiderate in five times refusing a patent—(if a just claim)—or else he was induced to grant it finally upon misrepresentation of facts—if an unjust one—and that he was, therefore, deceived; or upon a presentation of such evidence as he had overlooked in the first five investigations, and he was, therefore, not thorough but hasty; or else he is a weak and vacillating man, without sense enough to be so trusted, and should not be trusted with such power, a power exercised in this instance to the great detriment of the dearest interests of his country—its health and the protection of its babies.

There is but one other construction to be put upon this extraordinary, this most surprising decision; and there remains but one thing to do. The first we will not hint at—tho' we have heard it more than hinted at;—the latter we suggest and here urge:

Congress must be induced to go to the bottom of the matter—by a reliable commission—and to annul or revoke the patent. To that end we suggest that Parke, Davis & Co. and other wealthy firms engaged in supplying the profession with this Godsend—now enjoined—antitoxin—should get up a monster memorial to Congress, setting forth the facts, and when they are known the patent will be seen to be unlawful; and a protest, signed by the profession of America, should accompany the memorial. Congress should be thus petitioned in the interest of humanity, and I do not believe there would be among the seventy thousand physicians of America, one dissenting voice.

The JOURNAL calls upon Parke, Davis & Co., the universally recognized allies of the legitimate profession of America to head this movement.

We are authorized by this house, meantime, to say that they will personally undertake with their own means to defend any and every suit, should any be brought, for using their diphtheria antitoxin, and many surely will be brought, so that our readers may rest secure in this assurance and go ahead with the life-saving methods put into their hands by this firm—public property, now patented to a usurper—a foreigner—by the noble Commisioner of Patents of the United States Government, in gross violation of every principle of right, justice, law and dictates of humanity, else through incompetence or malfeasance of office.

Messrs. Parke, Davis & Co. write us: "We have retained the services of the foremost patent lawyers in the United States, and we propose to fight the pretended monopoly to the last trench. What we fear in the meantime is not the enforcement of the patent, but rather the possible intimidation to which the manufacturers and selling agents of the Behring serum may resort in their effort to alarm the physicians and pharmacists who place implicit reliance on our Anti-Diphtheritic Serum. We therefore respectfully request that in any article you may write for the JOURNAL you will insert a paragraph assuring your readers emphatically that Parke, Davis & Co. will protect and defend them from any legal proceedings that may be brought as a result of their purchase, sale and use of our serum, we assuming the entire expense of such defense."

